# Developing a model describing voluntary residency attrition: a qualitative study

**DOI:** 10.1186/s12909-024-05223-6

**Published:** 2024-03-01

**Authors:** Astrid J. de Vries, Yvonne ten Hoeve, Debbie A. D. C. Jaarsma, Jan Pols, Jos J. A. M. van Raay

**Affiliations:** 1 Department of Orthopaedic Surgery, Martini Hospital Groningen, Groningen, The Netherlands; 2 Health Sciences – Nursing Research, University of Groningen, University Medical Center Groningen, Groningen, The Netherlands; 3 Faculty of Veterinary Medicine, Utrecht University, Utrecht, The Netherlands; 4 Center for Education Development and Research in Health Professions (CEDAR), Lifelong Learning, Education and Assessment Research Network (LEARN), University of Groningen, University Medical Center Groningen, Groningen, The Netherlands

**Keywords:** Postgraduate medical education, Attrition, Model development, Grounded theory

## Abstract

**Background:**

Many factors influencing residency attrition are identified in the literature, but what role these factors play and how they influence each other remains unclear. Understanding more about the interaction between these factors can provide background to put the available evidence into perspective and provide tools to reduce attrition. The aim of this study was therefore to develop a model that describes voluntary residency attrition.

**Methods:**

Semi-structured interviews were held with a convenient sample of orthopaedic surgery residents in the Netherlands who dropped out of training between 2000 and 2018. Transcripts were analysed using a constructivist grounded theory approach. Concepts and themes were identified by iterative constant comparison.

**Results:**

Seventeen interviews with former residents were analysed and showed that reasons for voluntary attrition were different for each individual and often a result of a cumulative effect. Individual expectations and needs determine residents’ experiences with the content of the profession, the professional culture and the learning climate. Personal factors like previous clinical experiences, personal circumstances and personal characteristics influence expectations and needs. Specific aspects of the residency programme contributing to attrition were type of patient care, required skills for the profession, work-life balance and interpersonal interaction.

**Conclusions:**

This study provides a model for voluntary resident attrition showing the factors involved and how they interact. This model places previous research into perspective, gives implications for practice on the (im)possibilities of preventing attrition and opens possibilities for further research into resident attrition.

**Supplementary Information:**

The online version contains supplementary material available at 10.1186/s12909-024-05223-6.

## Background

Attrition of medical residents is of significant concern, having a negative impact on the personal, residency program and societal level (e.g. financial consequences, loss of healthcare professionals) [1–5]. Much research from recent decades has focused on attrition, and many factors that might play a role in attrition have been identified in literature. A poor work-life balance, female gender, fear of unemployment, lack of job autonomy, job content other than expected, poor skills performance and lack of social support at work may be associated with attrition, as demonstrated by several survey-based studies [3, 4, 6–10]. Three studies investigated the underlying reasons for residency attrition more in depth, using a qualitative approach [1, 5, 11]. The studies were all conducted among surgical residents who expressed concerns about training progression. Requirements were felt as box ticking or excessive. In addition, residents felt undervalued, experienced competition amongst residents, and observed clinical duties being prioritised over educational activities. Also, difficulties with supervisors, perceived quality of supervision and a perceived negative atmosphere, including bullying, came up as contributing factors. Worries about future roles and a scarcity of role models for better work-life balance likewise contributed to attrition [1, 5, 11].

Even though these studies provide much insight into the reasons related to attrition, it has not yet led to a reduction of the problem; despite several efforts, residency attrition rates remain substantial with 11% in hospital-based specialties and between 18—30% in general surgical residents [4, 12, 13]. This may be due to the fact that the main factors found in previous studies are part of the professional culture—including organisational structure and learning climate—which are difficult to change, but another explanation may be that essential knowledge necessary to understand and ultimately remediate attrition is still lacking. This is plausible since the available literature is mainly inventorying and descriptive in its studying and unravelling of contributing factors or themes. Only two studies were found that used a different approach, not identifying separate factors but using a model or a theory to explain attrition. Based on the available literature and theories of attrition at high school and college, Cusimano (1999) described attrition as a longitudinal process in which the background characteristics of an individual influence how that person interacts with the professional environment – which in turn leads to certain educational and attitudinal outcomes that ultimately determine whether an individual either chooses to remain in training or quit [14]. Contessa (2011) aimed to investigate the fit of surgical resident morale with the Menninger Morale Curve and the implications for attrition [15]. This theory identifies four psychological crisis periods when entering a new life situation: crisis of arrival, crisis of engagement, crisis of acceptance, and crisis of re-entry. The authors found that the periods of low morale tend to correspond with the moments in time residents are most at risk of quitting. Despite their relevance, there is an important limitation of these two studies for the purpose of explaining and understanding attrition. Both studies are theory-based, looking at whether the existing theory provides an explanation for parts of what happens in practice. To our knowledge, no study has focused specifically on residency attrition the other way around, building a model based on what actually happens in practice. Filling this gap in our knowledge is important to understand what makes a resident ultimately decide to quit and shed light on the (im)possibilities of prevention.

The aim of this study is to develop a model that describes voluntary residency attrition in the Netherlands using a qualitative approach. With this model we strive to give new insight in attrition that can be helpful in understanding why and how certain previously identified factors contribute to the decision to quit, thereby placing the available evidence into perspective and potentially providing tools to lower attrition rates.

## Methods

### Design

The study has an exploratory design, using semi-structured interviews for data collection. A constructivist grounded theory approach was used to develop a model ‘‘grounded’’ in the interview data. Fundamental elements of the grounded theory approach include an iterative process of data gathering and analyses, theoretical sampling, and using constant comparisons during data analysis [16].

### Setting

Our study focussed on former orthopaedic residents in the Netherlands. The programme starts with 1.5 years training in general surgery, followed by four and a half years of specific orthopaedic surgery training [17]. The activities of residents include outpatient control visits, surgeries, preoperative and postoperative care at the ward, and duties at the emergency department. Review meetings between residents and programme directors are scheduled every three months during the first year, every six months in the second and third years, and once a year in the last three years of residency training [17]. This programme is conducted in eight educational regions in the Netherlands, each region is led by a programme director. Twenty-one percent of all residents starting the orthopaedic residency programme between 2000–2021 were female and the average attrition rate in this period was 12%, with 27% of female residents and 7% of male residents quitting (data from the Registratiecommissie Geneeskundig Specialisten (RGS), retrieved 1-Apr-2021). Prior to their acceptance to specialty training, most of the residents work as a PhD candidate or a Doctor Not in Training (DNIT). As a DNIT one gains experience in the work field which is usually the specialty of first choice [18].

### Participants and ethical considerations

We included a convenience sample of former orthopaedic surgical residents who dropped out between 2000 and 2018 (no age limit; all former residents are above 18 years old). They were contacted by an email detailing the study’s aims and methods, with an attached participant information letter and consent form. Programme directors were asked to address the mailing to the relevant former residents in their educational region. They did not know who agreed to participate because former residents were requested to contact the independent researcher and interviewer (YtH), who is not connected to the orthopaedic training programme or to any orthopaedic department. The study was approved by the Ethical Review Board of the Netherlands Association of Medical Education (2019.2.16 and 2018.6.5). Signed informed consent was obtained from all participants.

### Data collection and analysis

Semi-structured interviews were conducted between November 2018 and October 2019 using an interview guide (Additional file 1). All interviews were digitally audio recorded, transcribed and pseudonymised. Following a constructivist grounded theory approach, we consecutively applied open, axial and selective coding [19]. In all stages of coding, constant comparative analysis was used, a fundamental process in a grounded theory approach. Data were constantly compared with other data in the same interview and between interviews. Moreover, the iterative nature of the grounded theory method was used by performing data collection and data analysis simultaneously. Early analytic insights and conceptual ideas obtained from the first interviews were used for further data collection. Data analysis was supported by ATLAS.ti (Atlas.ti GmbH, Berlin). Two researchers (YtH and AdV) separately open-coded the first four interviews. Differences and similarities in coding were discussed and codes were added, renamed, merged or deleted. The first four interviews were re-examined with the resulting coding list. Axial coding resulted in categories of thematically related codes [20, 21]. Selective coding was used to establish relationships between the categories in order to gain insight into the interaction of factors leading to attrition. The resulting model and its underlying analysis were discussed among the research team. Analytical thoughts and ideas were captured in memos throughout the analysis.

### Research team and reflexivity

It is important to take into account the different roles and positions of the members of the research team [19]. AdV is research coordinator at an orthopaedic department, YtH is a senior researcher in health sciences, JP is a non-practicing physician and senior medical educational researcher, DJ is a professor and research leader in (veterinary) medical education, and JvR is an orthopaedic surgeon and programme director. The significance of their background for analyses, conclusions and discussion were regularly discussed during team meetings.

### Trustworthiness of the study

To guarantee the rigor and trustworthiness, this study adhered to the criteria proposed by Lincoln and Guba (1985) in terms of credibility, dependability, confirmability and transferability [22]. Data credibility was established by selecting an appropriate method for the data collection (a semi-structured interview guide) and by the researchers who analysed the interviews being familiar with the context of residency training. Dependability was ensured by describing the data analysis in detail and providing direct citations to reveal the basis from which the analysis was conducted. The researchers coded the interviews independently from each other. The confirmability and consistency of the analysis were established by holding meetings to discuss preliminary findings, where codes and themes were discussed until a consensus was reached. This procedure was maintained during the entire coding process. To enhance the transferability of the findings a description of the context, selection of participants, data collection and process of analysis is provided.

## Results

**Fig. 1:**
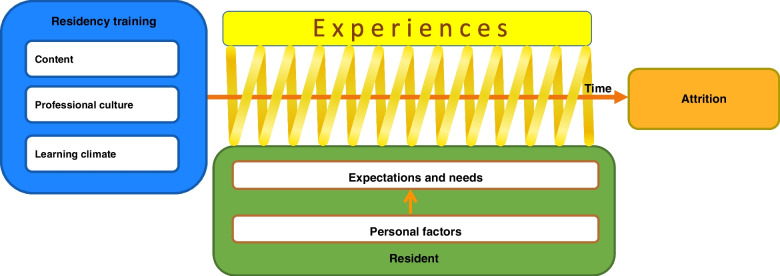
Model of voluntary residency attrition

After a short description of the characteristics of the participants in the study, the aspects of the ‘residency training’ as discussed by the interviewees are presented in this results section; with ‘content’, ‘professional culture’ and ‘learning climate’ being the main topics. Subsequently, the ‘resident specific aspects’ are presented, with ‘expectations and needs’, and ‘personal factors’ as the main elements. After a description of the ‘cumulative experiences leading to attrition’, ultimately the ‘model of residency attrition’ is presented (Fig. 1). The last part of the results section is the ‘emotional impact’ that accompanies residency attrition. Quotations are used to substantiate the results.

### Background characteristics

A sample of 18 former orthopaedic surgery residents participated in this study. Thirteen participants were female (72%), which reflects the almost-three times higher percentage of females dropping out (27% of all females versus 7% of all males) (based on data from the Registratiecommissie Geneeskundig Specialisten (RGS), retrieved 1-Apr-2021). Four dropped out somewhere along the 1.5 years of general surgical training, five in their first year of orthopaedic training, seven in their second year and two in their third year. Two participants switched to general surgery, six to general practice, four to a career in industry or policy, five chose another specialty (other than general surgery or general practice), and one was still exploring options for a future career. The interviews took place on average five years after discontinuing the training (range 1 month–12 years). Thirteen interviewees dropped out voluntarily, three others dropped out ‘voluntarily’ but took the decision after having received repetitive negative feedback. One case we consider similar to a voluntary drop-out is that of a resident for whom the decision was taken by the programme director: the resident was already having serious doubts and felt relieved that the decision was taken for him.

*Resident 14: For three years I had the chance to take the decision myself, and in the end it was taken for me. I just couldn’t bring myself to do it. When it was decided for me, things became easier.*

In another case the programme director forced the decision, resulting in a lot of resistance and disbelief by the resident. This resident was not included in the analysis, as we believe a forced drop-out is significantly different from the other interviewees. Hence, in total 17 interviews were used for the analysis.

### Residency training

Analysis of the interviews shows that orthopaedic residents distinguish two aspects of the context of their training: the content of the profession and the atmosphere they work in. On the latter, two different yet related aspects can be distinguished: professional culture and learning climate.

### Content

The interviews revealed two aspects about the content of residency training: type of patient care and skills required to provide this care.

#### Type of patient care

The type of patient care is strongly associated with the category of patients one faces. Patients in orthopaedics are described as mostly having serious, burdensome and painful problems but not being severely ill. The interviewees feelings of providing care to these patients ranged from very comfortable to feeling it was too predictable. In the latter case they struggled with the limitations of orthopaedic patient care, citing the limited time per patient and the diagnostic and therapeutic possibilities.

*Resident 4: Even the standard knee is never standard, so in orthopaedics I found this a bit short-sighted. It was either the meniscus or the cruciate ligament. Operate or not operate, no, just go to the physiotherapist, we cannot help you. It was a shot, physical therapy, surgery or nothing. And so there was no time for a more thorough examination, to see how the patient is really doing.*

#### Skills

Both the technical/manual skills one needs to perform surgeries, and the more generic skills were mentioned, like the ability to make quick decisions and take on responsibilities. Residents discovered that the required skills did not always suit them. For example, having to take on a lot of responsibilities and being the one to make important decisions was very satisfying for some, while others were not comfortable with it at all, as the quote of Resident 13 exemplars. Concerning the surgeries, some experienced performing them as very enjoyable and an interesting part of the job while others did not like to perform surgeries themselves, having the feeling that they lacked skills to operate, leading to insecurity (Resident 17).

*Resident 13: I’m the perfect second violin. I don’t just sit on the bleachers. I noticed I was getting more responsibilities in my duties and people did think I could handle it. And I did, but with great difficulty.*

*Resident 17: Once I actually got to operate, I really didn’t like it. […] After you’ve made certain progress in your training, you have to dare cut into things, you’ve got to have the nerve to operate. I wasn’t top-talent, some people can practically operate just by reading how to do it […]. But I didn’t feel too good when operating.*

### Professional culture

Regarding the professional culture of orthopaedic surgeons, interviewees mentioned in particular the high workload and the scheduling of the working hours and shifts, and how this negatively affected their personal life and work-life balance. But the hard-working conditions also caused a feeling of togetherness with their colleagues. The masculine work environment was mentioned as well, where the no-nonsense attitude was perceived as pleasant or perceived as little room for personal attention or to share feelings of insecurity. Some felt the disadvantage of being a woman and needing to step up even more than their male peers.

*Resident 13: You had to work really hard in orthopaedics, really hard, lots of outpatients, not a lot of supervision. Also among my peers I noticed: working hard was the credo. If an outpatient appointment was cancelled or you were done early in the OR, you always had to fill the time with something else. There was even little time for administration …, you had to do that in your free time.*

*Resident 16: I think that, as a woman, you had to bring in a little more. Maybe it does count, especially so that they don’t see your soft side or your vulnerability, you basically have to play the game and act tough.*

### Learning climate

The learning climate can be defined as the physical and psychological environment including how trainees perceive the overall teaching and learning conditions [23, 24]. What the residents said about the learning climate relates to three topics: availability of supervision, differences among supervisors, and the interactions with other residents in training.

#### Availability of supervision

Residents mentioned that the high workload of orthopaedic surgeons has direct consequences for the availability of supervision and the need to be (very) self-reliant. Residents also felt that during shifts the threshold to contact a supervisor was high, and that by asking for supervision they risked negative consequences.

*Resident 7: I got assigned to trauma duty, and the first time in the trauma room there was severe injury, orthopaedic injury […] the trauma surgeon said, where is your supervisor? So I called again, […] and in the end he did come […] But thereafter the supervisor started asking the older-year residents how is she (e.g. resident 7) doing, is she really that insecure, and you should keep an eye on her because, well, this isn’t good. And then I thought, was that so bad?*

#### Differences among supervisors

Residents noticed differences among supervisors on how to perform in orthopaedic patient care and in ways of supervising that hindered their ability to learn. While several supervisors were perceived as pleasant and supportive, residents also had negative experiences, leading to feelings of an unsafe learning climate and lack of trust. Supervisors’ personal opinions on how to do things and how to treat patients were intertwined in the supervision which led to an experience of disapproval when they did things differently, and feeling the need to conform with the supervisor’s standards.

*Resident 5: You learn something from everyone. But sometimes it’s confusing. Because if you’re just trying to learn something well, you have someone saying we always do it this way, and someone else saying we always do it that way. And one day you do rounds with this one, tomorrow with that one. So it’s very hard to have continuity.*

*Resident 10: It wasn’t a safe learning climate. So if you made a mistake, you were severely punished.*

*Resident 12: In every conversation – even if it was very short – I liked having real contact. […] Make a joke, laugh with people. Share something personal. […] Put people at ease. That gave me pleasure in my work […] But I was criticised for it: ‘No, you have to be much more business-like’.*

#### Other residents

In addition to supervisors, fellow residents also played a role in the experienced learning climate. The interviewees distinguished between residents in the same training phase and more experienced residents, who had a role as junior supervisors, helping them in unpredictable situations and with reflection on experiences. Their peers from the same training phase were important to share experiences with and to feel part of a team, but residents mentioned a competitive atmosphere as well.

*Resident 17: It is very much performance-related, everyone wants to be the best, everyone wants to be in the operating room. Plus you have to do research in the evenings or at night. And then you have to say to everyone: last night I worked until 3 AM, and I loved it! Egging everybody on!*

### Resident specific aspects

We have explored the range of residents’ experiences regarding the content as well as the professional culture and learning climate. The analysis of the interviews also sheds light on what influences their experiences: residents’ expectations and needs, and personal factors.

### Expectations and needs

The interviewees were often explicit about experiences that did not match their expectations and needs with regards to the content of residency training, professional culture or learning climate. The labour market was also mentioned in the interviews; the poor career prospects for orthopaedic surgeons in the Netherlands contributed to individuals’ decision to quit.

*Resident 4: Chatting a bit with the patient and hearing the story and more than just that knee and just that hip, I liked that. And then, well, you know, you had to actually perform orthopaedic surgeries. I did like the detailed work, hands, feet, but hips and knees … Especially hips, I hated that.*

*Resident 1: And I’m actually on the cautious side and am not quick to think that I know everything and this is the way we’re going to do it. I am not that directive in my communication style, I think I had more of a need to work under a supervisor.*

*Resident 6: There was a lot less space for developing in other areas. […] I’m not someone who wants to be able to do just one thing well, I want to be sort of a Renaissance man … And now there is the fact that I’ve been following the training for six years at that pace in order to become an orthopaedic surgeon and that I also have a total lack of security as to whether I would find a job doing that.*

Different expectations and needs result in different experiences with the same situation. An example is the difference between residents in experiences with the high workload, long days and shifts, as the following quotes of resident 17 and 11 demonstrate.

*Resident 17: It was hard work, but I was single and had no other obligations, and we also did fun stuff outside work.*

*Resident 11: And I really found it to be an awful shift. Then I thought: I just don't want to do this anymore. I felt like a failure in orthopaedics. I am a bad mother, I'm never home (…) And then you start thinking: what kind of mother am I, what role am I taking upon myself? It was not orthopaedics in itself, in fact I would still choose it.*

### Personal factors

Individual expectations and needs are influenced by several personal factors. Personal circumstances e.g. being single, running one’s own business, becoming a parent were mentioned (as was the influencing factor in the example under ‘expectations and needs’). Another contributing factor is the influence of personal characteristics, like being a perfectionist, having a more passive personality, or feeling insecure easily. Residents also reflected on their previous clinical experiences and the extent to which they were prepared for the actual work, with those who had worked as a doctor not-in-training at an orthopaedics department commented that the majority of their time at the ward was not good preparation for the actual job of being an orthopaedic resident and surgeon.

*Resident 4: I also found the hospital to be a very demanding environment. The operating room too, you know, it has to be perfect. And I’m already a perfectionist to begin with.*

*Resident 7: And that doctor not-in-training period can be valuable, because you develop as a doctor. And during that time you should actually be exposed to activities that belong in the life of the medical specialist that you would like to become. […] For me this was not well-balanced. Because nonsurgical work is very different work. It is more like a confirmation that you have really good organisational talent plus communication skills and empathy skills, [are] good at teambuilding and cooperation and that you meet your appointments, for example. But that doesn’t mean you’re technically good at operating.*

### Cumulative experiences leading to attrition

Above we described the separate aspects related to the residency program and the resident, but our interviewees described attrition as a result of a series of cumulative experiences, where the negative experiences—not meeting individual expectations and needs – become more dominant in time. The experiences were reflected on by the residents, in the process of changing expectations and needs, which in turn influenced how the next experience was perceived. This is illustrated by this quote from *Resident 17*.

*Resident 17: Yes, it’s a bit of a process. A process of finding out, wow the clinic is not that interesting, very often you reduce the pain maybe just a bit, sometimes you don’t. In many operations I felt like do I actually have to do this, it is super fun, super good, technical, but to what degree are you really helping this person? […] So I ended up a bit in some sort of downward scale.*

For most residents, the reason for leaving specialty training was multifactorial – multiple aspects of residency training contributing to the decision to quit.

*Resident 12: For me there is not one single reason, but if I have to describe it and summarise it in one sentence? I think that in terms of content it is often enjoyable, but then there is all that comes with it in terms of people and the hospital, which doesn’t fit with me at all, as a person or as a doctor.*

### Model of residency attrition

Figure 1 shows the model of residency attrition that was developed based on the interview data. All residents are confronted with the context of the residency training, which is the content, professional culture and learning climate, and they experience this context in a diversity of ways. The interviews show that the way residents perceive and reflect on these experiences results from certain resident-specific aspects: their expectations and needs, plus personal factors. Expectations and needs change in the course of a residency training programme. This change is not only a result of the increasing number of experiences as a resident, which can influence the expectations and needs either positively or negatively, but also of the personal factors that may change over time – for example, a starting resident may be single but later becomes a parent. This changes the resident’s personal circumstances dramatically, with the ensuing altered expectations and needs. For residents who drop out, the experiences and changing expectations and needs led to cumulative negative experiences, doubts about the continuation of their training, and ultimately the decision to quit.

### Emotional impact

In addition to the model describing voluntary attrition (presented in Fig. 1), the interviews revealed a substantial emotional impact of quitting residency training. For most residents, the decision to quit the residency training was the result of a long personal process of uncertainty and doubt. They commented on the influence their decision had on the workload of their colleagues, which influenced their decision-making. Having to justify to their personal environment about quitting orthopaedic residency training and opting out of a career as a medical specialist was seen as a loss of status. Also the period after the decision is made was experienced as emotionally demanding. All interviewees reflected a lot on the impact of quitting. This demonstrates that also for residents who dropped out voluntarily, a decision to quit is not easily taken and processed. Even though they believed the decision to quit was unavoidable and the best one, their reflections are often associated with a sense of failure and/or feelings of guilt. Severe grief is also experienced by the residents after quitting because they had to let go of the image or dream they had for years.

*Resident 5: For me it’s been a very long process of doubt, because stopping with the training is not a small thing, it has all been quite profound […] Yeah, because it is a very long process where you have to really think, you know, I have doubts, do I really want this, am I going to really do this? Am I going to spend my entire life the way it is now without knowing what I’m getting in return? And that does have consequences.*

*Resident 6: I do know that at a certain point I told my father yes, I don’t know if I want to become a medical specialist, maybe just work a little less and become a general practitioner. That he said be careful, because before you know it you may end up being something like an insurance doctor. You know, this confirms there is a sort of degradation, a sort of hierarchy in importance, so to speak. So yes, these were of course all things that were playing a role. So I would have to let go of that prestige aspect.*

*Resident 4: I never wanted to do it any other way. And still, you know, when I stopped I really had a period that was some kind of grief processing. It sounds heavy […] you have to say goodbye to the image you always had of yourself.*

## Discussion

We started this research with the intention to develop a model that describes voluntary residency attrition in The Netherlands, to better understand how the decision to quit was made. Our results show that experiences with residency training differ between individuals, and that individual expectations and needs are important modifying variables. Expectations and needs are in turn influenced by personal factors, like previous clinical experiences, personal circumstances and personal characteristics. Experiences may differ in time as expectations and needs can change, not only because of the increasing number of experiences as a resident, but also because personal factors like personal circumstances may change over time. Our study showed how multiple different factors act cumulatively, leading to doubt and ultimately the decision to quit. This decision is accompanied by substantial emotional impact.

Our research shows that the interaction between expectations, needs and experiences among our interviewees leads to an accumulation of negative experiences, often with more than one aspect of residency training which led to feelings of doubt and ultimately to the decision to quit. This concords with the results of Liang et al. [11], who described that multiple factors act cumulatively until a certain threshold is reached. With this knowledge one can understand that the wide range of reasons for attrition found in the available literature all have their place in attrition but are not explanatory on their own. An example is the frequently cited poor work-life balance. Despite that all residents are faced with similar high job demands with long working hours and shifts, the majority of residents do complete residency training successfully. So, this aspect is not a sole determinant of attrition. But when childcare responsibilities come into play (changing needs), combined with resident specific variables like a perfectionistic nature and increasing fear of unemployment after ending the training program, this may after a certain period of time lead to a threshold being reached, resulting in attrition. Several factors contribute, but our interviewees showed that a cumulative effect was needed to result in attrition, since the decision to quit is very difficult and a result of a prolonged awareness process accompanied by feelings of grief, guilt and failure. These findings might help explain the findings of Bustraan et al. (2019), that a relatively large portion (25%) of residents drop out late (in years 4, 5 or 6), which according to the authors indicates that it takes time to realise the mismatch between resident and residency programme [4]. Our results show that, next to changing personal factors, the relatively large numbers of late-leavers could very well be a reflection of the emotional difficulty of the decision.

The practice-grounded model we developed in this study fits well with the theoretical description of attrition provided by Cusimano (1999). This author described attrition as a longitudinal process in which the individual’s background characteristics influence how that person interacts with the environment [14]. One theory used by Cusimano was developed by Tinto (1977) and explains withdrawal or persistence in higher education by acknowledging that, to persevere, an individual must integrate in, and commit to, both the social environment and the academic domain [25]. Tinto already acknowledges that the dropout process is longitudinal, involving interactions between the individual and the academic and social systems [25]. The personal experiences with these systems change the commitment: negative experiences can lead to lower commitment, which could result in dropping out, while positive experiences can yield enhanced commitment and perseverance. The results of our study show a similar process of interaction between the individual and the two systems, where the social environment is reflected by the professional culture and learning climate, and the academic domain is reflected by the content of the profession. Tinto’s model [25], originally developed for higher education, holds for residency attrition too. Cusimano applied this theory to residency attrition and stated that leaving training may be seen as the result of adverse personal experiences in the overall culture of the institution (formal and informal). The adverse experiences Cusimano found in neurosurgery residents [14] are the excessive workload within the training programme – where the social and personal costs outweigh the intrinsic rewards associated with the training – combined with an underestimation of the residency’s needs [14]. These results are also part of the model we developed.

Specific aspects of residency training in the current study where a mismatch between expectations, needs and actual experiences was perceived were type of patient care, required skills, work-life balance and interpersonal interactions. These aspects contributing to attrition cover all facets of residency training and fit well not only with the theoretical model of Tinto [25] but also within the three purposes that Biesta and van Braak (2020) suggest to comprehensively describe the goals of medical education: professional qualification, professional socialisation and professional subjectification [26]. Regarding qualification – gaining knowledge, skills and understanding – in our study several residents reported not feeling competent to perform surgeries or being discontent with the narrow scope of qualifications needed for the profession. The domain of socialisation – the ways of being and doing, the norms and values and particular traditions of a professional group of people [26] – in our study is represented by the experience of a disturbed work-life balance due to the culture of working long hours and shifts, and by having difficulties with interpersonal interaction styles, which residents resist becoming part of and which cause friction or feelings of isolation. The latter domain of purpose, subjectification – which entails residents becoming free to have their own ideas, draw their own conclusions, and take responsibility for their actions [26] – likewise plays a role. Our interviewees reported not always feeling free or supported to present their own ideas or be autonomous in their actions, as the opinion of the supervisor can be judgemental and strongly dependent on their own preferences.

An important part of the model we describe is the role of individual expectations that change experiences. This concords with Abelson et al. (2018), who found that general surgery interns who had more realistic expectations of residency training and the accompanying lifestyle are more likely to complete the training [27]. This stresses the need to have relevant experiences with a specialty prior to joining the residency training programme. In the Netherlands most residents work as a ‘doctor not in training’ (DNIT) in the same specialty before starting their residency training. Bustraan et al. (2019) showed that residents with this experience mention ‘work content other than expected’ less frequently as a reason for quitting – 38% vs 58%. [4]. Still, over 60% of the Bustraan et al.’s respondents quit their residency training despite prior experience as a DNIT in the same specialty. Our study shows that working as a DNIT does not always yield relevant realistic experiences with work that are central to the specialism: DNITs mostly work on wards while orthopaedic residents and surgeons usually work in the outpatient clinic and operation room.

### Implications for practice

In this study, we showed that none of the many factors found in previous research individually lead to the decision to drop out of residency training, but that it is cumulative and multifactorial in origin. The consequence of this finding is that our advice for practice does not focus on individual factors, but on the context in which those factors play a role.

First, programme directors should be aware of the importance of realistic expectations about the speciality among future residents, based on sufficient but also relevant experience in the work field. The latter is important since former residents indicated that even having worked as a DNIT is not good preparation for the actual work and field of work of the profession. We therefore suggest a substantive review of the content of the DNIT programme, especially since programme directors prefer to select those residents they know and have worked with [28].

Another aspect that appears to be important in the decision to leave residency is the professional culture – hard work, long hours, shifts, little attention for the individual or individual situations – that does not always meet the personal needs of residents, especially in terms of work-life balance. Considering the ongoing developments in patient care (like higher turnover rates) and the changing perspectives of younger generations towards work and work-life balance, we wish to encourage departments to look critically at their professional culture and to evaluate whether it still meets the expectations and needs of the younger generations they are training – not only to prevent attrition but also for their own benefit, as several studies show that emotional distress and symptoms of burnout are a problem among medical doctors too [29–31].

A third practical implication of this research is the need for program directors to create a climate in which residents can safely express their concerns and doubts. Topic of conversation (in the periodical review meetings) should be the components depicted in the model: experiences with all aspects of residency training in relationship with possibly changing expectations and needs and personal factors. It is possible that more openness can help some residents to overcome their concerns and doubts, but it is still important to remember that attrition cannot always be avoided. A consequence of the longitudinal character of residency training is that career prospects and expectations and needs of residents may change over time. Hence a good initial fit between resident and programme is no guarantee of successful completion. Our study shows that getting older, and learning more about one’s personal preferences, but also live events like parenthood can change expectations and needs dramatically, resulting in a mismatch that originally was not present.

### Future research implications

The model we developed about resident attrition is an important step towards a more refined (middle range) theory. To develop this, various parts of our model need to be further developed with qualitative research, for example to further map residents’ expectations and needs, their mutual relationship and their occurrence over time. The interrelationships between the identified factors could then be tested quantitatively (with surveys) to determine their strength and importance for the decision to quit.

Considering our research aim, in this study only residents who (voluntary) dropped out were interviewed. A previous survey-based study demonstrated that almost 60% of surgical residents in training have serious doubts about the continuation of their residency program [32], and a survey amongst Dutch Orthopaedic residents showed that almost 20% had poor quality of life and almost 50% was not satisfied with their work-life balance [33]. As attrition rates found in literature are between 11– 30% [4, 12, 13], one can conclude that the majority of those residents in doubt or having problems with the work-life balance ultimately do complete their residency training program successfully. It would be interesting to study the experiences of these residents as well, to gain knowledge about what aspects—related to the resident or to the residency training program—make that one continues and successfully completes residency training. More insight into those aspects that work as motivators to overcome the doubts, leading to persistence to the training program, may provide relevant tools for the prevention of attrition.

Because of the authors’ familiarity with the setting, only former residents of one medical specialty (orthopaedics) in one Western European country were interviewed. That all our participants had a comparable context during their residency training was a strength of this study, as including residents from multiple specialties would have led to extra complexity and findings that are harder to interpret. We believe that the model we developed is likely to apply to different, non-surgical, specialities or other countries, considering that our practice-based model fits well with existing theories and descriptions of attrition, and similar aspects contributing to attrition are found in previous studies. Nevertheless, the actual transferability of the model should be topic of future research as well.

## Conclusions

Our study provides a model of residency attrition where a cumulative mismatch between individuals’ expectations and needs – influenced by personal factors – and the experiences with the content of the profession, the professional culture and the learning climate lead to doubts and ultimately the decision to quit. The decision to quit has significant emotional impact. This model places previous research into perspective, gives implications for practice on the (im)possibilities of preventing attrition and opens possibilities for further research into resident attrition.

### Supplementary Information

**Supplementary Material 1. **

## Data Availability

The datasets generated and/or analysed during the current study are not publicly available due to participant confidentiality as per consent obtained from participants. However anonymised and restricted interview transcripts are available from the corresponding author on reasonable request.

## Abbreviation

DNIT: Doctor not in training
