# Supplementary material for: Developing a model describing voluntary residency attrition: a qualitative study
Source: BMC Med Educ. 2024 Mar 1;24:221. doi: 10.1186/s12909-024-05223-6 (PMC10908006; doi:10.1186/s12909-024-05223-6)
Supplement: Supplementary file 1 — Supplementary Material 1. [file 12909_2024_5223_MOESM1_ESM.docx]

# Additional file 1

**Topic list of interview**

**Choice of orthopaedic specialism**

- What were the main reasons you chose the specialism of orthopaedics?
  - Did you consider other specialisms? Did you only consider surgery or also a nonsurgical or supportive specialism?
  - Did you also consider options other than medical specialist, such as general practice, social medicine or insurance medicine?
- Did you already have experience as a doctor not-in-training when you started the programme, or did you have any other type of clinical experience? Can you elaborate on this?
- Has your environment (family/friends) influenced your choice? If that is the case, how?
- What were your expectations before starting orthopaedic training (and based on what)?
- What kind of clinician did you want to be in your role as a doctor-in-training?

**Experiences during the orthopaedic programme**

- In general, what were your experiences during the orthopaedic programme?
  - Content / education / supervision / attitude of teachers / trust of teachers / trust in yourself
  - Did the expectations you had at the beginning correspond with the reality?
- What specific aspects of the program did you like or found instructive?
- Which factors within the program motivated you? What helped you?
  - Social contacts with other doctors-in-training? Procedures in the OR? Autonomy? Feedback and support? Science?
- What specific aspects of the program did you like less or found less instructive?
- What issues during your studies demanded a lot of energy from you (consumed all your energy)?
- How was assistance by the teachers organised?
- What was the atmosphere like in the programme’s group? And among the doctors-in-training?

**Quitting the programme**

- What made you consider to quit the programme?
- When did you start having doubts as to whether you should or wanted to continue with the programme?
  - Was there a main reason/trigger for you?
- What kind of help was offered the moment that doubts started?
  - From the teacher / fellow doctors-in-training / from the home front / psychological support / support from the central programme committee / study house / departmental head / academic hospital?
  - What do you believe should be different in the programme to avoid dropouts?
  - Do you have suggestions for improvement?

**Current function**

- What is your current function?
- How did you get to that function?

**Differences between current/completed program versus orthopaedics (if applicable)**

- What do you consider to be the biggest difference between your current/completed program and the orthopaedic surgery program?
  - content of the programme / environment of the programme / myself / changed home situation / …
